# Mental processes in professional football players

**DOI:** 10.3389/fpsyg.2024.1428892

**Published:** 2024-09-04

**Authors:** Johan Grønset, Martin Langagergaard, Stig Arve Sæther

**Affiliations:** ^1^Department of Sociology and Political Science, Norwegian University of Science and Technology (NTNU), Trondheim, Norway; ^2^Performance Psychology Consultant, Learn To Improve, Aalborg, Denmark

**Keywords:** mental processes, mental toughness, arousal regulation, self-confidence, professional footballer, sports psychologist

## Abstract

**Objective:**

Clear connections have been found between mental processes and performance in elite level football. Yet, few studies have investigated how professional football players’ experience the influence of mental processes on performance.

**Method:**

This study used a qualitative research design and in-depth interview of six Norwegian professional football players at elite level with a mean age of 28, 3 years and represented five professional clubs. The aim of the study was to investigate how they perceived the importance of arousal regulation, mental toughness, and self-confidence, and that of the sport psychologist in developing these skills.

**Results:**

The results revealed a complex and multifaceted link between mental processes and performance. All players, demonstrate a conscious awareness of how mental processes influence their performance. The use of a sport psychologist in working with mental processes emerges as a crucial factor for developing their mental skills. Another important aspect is the need for increased knowledge about mental training’s effect in elite football. Mental toughness emerges as the most significant mental process for players’ performance because it makes them capable of coping with challenging situations and periods.

**Conclusion:**

This study shows that mental processes are important for performing, both related to arousal regulation, and self-confidence, and especially mental toughness. However, mental toughness was also considered a product of age and experience, where older players tend to have experience of more situations that enable them to handle adversity better than younger players. Interestingly, the youngest players seem to be most aware of the use of a sports psychologist.

## 1 Introduction

Professional sports require the performing athletes to possess a range of skills. One of the most challenging set of skills to develop are the mental skills to cope and develop as an elite athlete (Konter et al., 2019). Compared to non-elite athletes have elite athletes been found to have higher score in self-efficacy, emotionality, present fatalistic time perspective, past positive time perspective, and openness to experience (Mitic et al., 2021). A vital part of developing mental skills is the mental processes which refer to internal, invisible, activities in our minds. These include thinking, reasoning, and problem-solving, and form the basis of our actions, decisions, and feelings (both conscious and unconscious), which govern the person’s perception of themself and their surroundings, and processes that form the basis of their emotions and desires (think, understand, learn, remember, and make decisions) (Ivarsson et al., 2020). Research has shown that mental processes (and the mental skills learned in the process) are important contributors which not only directly influence their ability to perform at their best (Nesti, 2010), but also indirectly by optimising other skills required to succeed at the top level (Williams et al., 2020).

Sports psychology research has a wide range of both qualitative (Gould et al., 2002) and quantitative (Mitchell et al., 2014) studies on elite sports. However, there is a narrower selection of both qualitative and quantitative studies on professional football players’ experiences (Ivarsson et al., 2020; Jordet, 2019), despite several quantitative studies at the junior level (Benítez-Sillero et al., 2021; Forsman et al., 2016; Saward et al., 2020), showing that older junior players can withstand tougher mental stress. The conscious and active use and development of mental processes is necessary to better handle poor performances (Saward et al., 2020). Furthermore, footballers who have become professionals had clearly prominent psychological skills at a young age, such as increased self-confidence, commitment, and ability to handle pressure (MacNamara et al., 2010; MacNamara and Collins, 2011; Rye et al., 2022).

Three mental skills which have been shown to be important and partly connected to performing well in professional football are mental toughness, arousal regulation, and self-confidence (Williams et al., 2020). Mental toughness is the most complex phenomenon, and is related to endurance, continuity, and motivation (Jones et al., 2002). It is the ability to handle poor performance and stressful situations such that it does not negatively affect performance. According to DeWiggins et al. (2010) mental toughness can be described as the ability to be focused, composed, and safe in stressful situations. Some characteristic features of people with high mental toughness are their ability to assess and reflect on their own performance, high self-esteem (self-confidence), and a good ability to handle adversity (Wieser and Thiel, 2014). The literature reveals clear connections between mental toughness and performance, and the importance of this mental process as a psychological aspect (Thelwell et al., 2005; Coulter and Thelwell, 2019). Thelwell et al. (2005) found that soccer players suggested that players who demonstrate attributes resembling a high level of self-belief and an ability to cope with the internal and external pressure that elite sport places on the performer, tend to be perceived as mentally tough. Furthermore, Miçoogullari and Ekmekçi (2017) found a positive correlations between psychological skills training and mental toughness among professional football players, in a sixteen weeks training program. Statistically significant differences were found between pre-test and post-test values which were been confidence, constancy, control, mental toughness, self-acceptance, positive relations with others, autonomy, and psychological well-being.

The sports psychologist is an important actor for developing psychological skills in professional football (Nesti, 2010). However, they face four main challenges in delivering in elite and professional sports: congruence (operating authentically, and in line with their personal philosophy and with their chosen methods), having a broader role (e.g., managing multiple relationships), influence of elite sport cultures, and surviving and thriving (McDougall et al., 2015). Similar challenges have been found in football (Kremer and Marchant, 2002; Jordet, 2019; Feddersen et al., 2023).

If the athletes are doing an individual or team sport, they are a part of a team of performers and influence each other’s development and performance. The individual footballer must also function as part of a performing team. Langagergaard (2017) pointed in this regard on the importance of establishing conditions for a common language that forms the basis for a group to develop a performance culture together. The concept is a cyclic, temporal, and transitional approach, and serves as common language of reference regarding performance psychology (described further in Langagergaard, 2017). The “four phases” consists of: “pre-phase” (phase 1), “during-phase” (phase 2), “post-phase” (phase 3) and “transfer-phase” (phase 4). The first the “pre-phase” (phase 1), which is a preparation phase for the individual (and/or team preparation) at the practical, physical, and psychological levels; essentially, this phase is “getting ready to perform”. In football, phase 1 can be understood as match day preparation, locker room time, and warm up. Phase 2, “during-phase”, refers to the duration of time where an actual performance is taking place; in football, it can be translated into the first or second half of the actual football match. Phase 3 is the “post-phase” and is characterised by evaluation. It involves processing impressions and to some degree being able to “learn & close” the performance over time and then moving onto to phase 4. Phase 4 is the “transfer-phase”, characterised by resetting, recover and “switching off” from the performance mode. This four-phase approach takes the perspective of the individual (player) and the use (aware/not aware) of well-known mental training techniques within three domains: intend, attention, and intensity, i.e., motivation, goalsetting, arousal regulation, focusing, mental routines (pre/during/post), and mindfulness.

Here, our intention is to dive into professional footballers’ experiences of the influence of mental processes on performance in a Norwegian context which lacks research and how they have developed these processes with the help from a sport psychologist. We therefor interviewed six Norwegian professional football players to seek to gain insights into professional footballers’ experiences of and around the relationship between mental processes and performance, especially those related to mental characteristics such as arousal regulation, mental toughness, and self-confidence. Furthermore, we explore the players’ perception and experience of what Langagergaard (2017) has described as a common language related to mental skills and processes while working with a sports psychologist in both their current and earlier clubs.

## 2 Methods

The study investigates how professional football players perceived the importance of the mental processes and use of a sport psychologist, and how these influence their performance as players. The study focuses on the everyday interactions between individuals. Further, the meanings with these interactions are managed and transformed through peoples’ interpretative processes. Hence, we have taken a social interactionist ontology stand and utilise an interpretivist approach (Markula and Silk, 2011; Wahyuni, 2012). The study was approved by the Norwegian Centre for Research Data (reference number 410262) prior to the data collection.

### 2.1 Participants

All interviewed players were professional football players at the elite level (one player played level 2), had a mean age of 28,3 years (SD = 4.3), and represented five professional clubs. These players had a long experience in professional football and were recruited by the first (3 players) and last authors (3 players). Both these researchers have a network of acquaintances as they were a part of a professional football academy and a researcher of professional clubs for a decade, respectively.

### 2.2 Interviews

The interviews took place either at the players clubs or digitally, and lasted between 23 and 50 min, with an average of 39 min. Three interviews were conducted face to face, while three was done digitally (Zoom). Each participant was interviewed only once and by the same interviewer. All interviews at the clubs were conducted in a quiet area which was chosen by the participants, with only the interviewer and participant present. The interviews used a semi-structured approach, as detailed by Brinkmann and Kvale (2018), and were audio recorded and later transcribed verbatim (see Appendix 1 for the interview guide). To ensure confidentiality, all participants were anonymised in the transcriptions and their pseudonyms were used (see Table 1).

**Table 1:** Description of the participants.

Player ID	Age group	Experience with a sport psychologist	Playing level	Other information
Tommy	20–24	Access to SP in club, no personal relationship with SP	Norwegian elite level (experience from different elite level clubs)	Age-specific national team matches, no education.
Chris	30–34	No access to SP in his club (external lectures), no personal relationship with SP (lack of time with SP)	Norwegian elite level 2 (experience from different elite level clubs)	Been in same club during his professional career. Same club as P4. Two non-sport related bachelor’s degrees
Oscar	30–34	Access to SP in shorter periods, personal relationship with SP	Norwegian elite level 2 (experience from different elite level clubs)	Sport related master’s degree
Peter	20–24	No access to SP in his club (has reach out to a SP), had personal relationship with SP	Norwegian elite level 2 (experience from different elite level clubs)	Age-specific national team matches, same club as P2, non-sport related education
Frank	25–29	Access to SP, personal relationship with SP	Norwegian elite level (experience from different elite level clubs)	Non-sport related bachelor’s degree
Oliver	30–34	Access to SP, personal relationship with SP	Norwegian elite level (experience from different elite level clubs)	Age-specific national team matches, team captain, non-sport related bachelor degree, current student

Adopting a social interactionist ontology and interpretivist epistemology enabled us to frame our interviews as a relational space, meaning that both the participants and interviewer could explore themes together and co-construct knowledge (Markula and Silk, 2011; Wahyuni, 2012). The interview guide used to structure the interview process included the following topics: *arousal regulation, mental toughness,* and *self-confidence* (see Appendix 1 for the complete interview guide).

### 2.3 Data analysis

We examined all interview materials using a six-step thematic content analysis developed by Braun and Clarke (2006) and Braun et al. (2019). First, the first author transcribed, read, and re-read the data. Second, the same author generated initial thematic codes by inductively analysing the data (for example, ‘mental match preparations’). Third, these were then presented to the second and third authors, whose role was to act as ‘critical friends’ who reviewed and challenged the first author’s descriptions and reasonings for these initial codes. All authors discussed how the findings should be categorised and structured into higher-order themes (for example, ‘Understanding and use of mental processes’). Fourth, all authors then jointly elaborated on the analytical themes and returned to the raw interview data to clarify questions (for example, what relationship did the players have while working with a sport psychologist). Fifth, the sub-themes and final categories were reviewed and refined. The final step combined the analytical and categorisation processes. Hence, the authors often went back to the categories and raw data to ensure that the content was fairly and accurately based on what had been defined during the report writing process.

## 3 Results

The results are centred into the following topics: understanding and use of mental processes, mental toughness, arousal regulation, self-confidence, and the role and use of a sport psychologist with respect to performance.

### 3.1 Understanding and use of mental processes

The fluid concept of mental processes described in the research literature was also reflected in the players’ answers, which ranged from general thoughts on the use of, for example, inner dialogue to more specific thoughts related to toughness, as described by Frank below:

Frank: Mental processes is for me, it really means how much you can stand to actually endure. It could be if you get injured, then you have to deal with it and come back from it. How do you deal with it if you have a bad game or a good game, are you able to basically reset from both of those for the next practice and the next game[,] or are you affected by that?

Still, the players valued the importance of focusing on these mental processes continuously and dynamically. This is in line with earlier research on a performance culture with changing situations and difficult periods (MacNamara et al., 2010; Thelwell et al., 2005), where the players are expected to perform and handle pressure (Dodd and Newans, 2018; Williams et al., 2020).

Frank: Actually, the most important thing is to try to have a continuous process. It cannot be the case that you screw it up, and then suddenly an important match comes up and you have to focus a lot on that. Then I think it goes against its purpose.

However, some players described the process of going through the different challenging thoughts, thought by thought so to say, to be able to address them instead of ending in, as what one of the players describes, a black hole:

Tommy: Often it is also like this if there are periods when things are a bit tiring, such as if you are injured, you see someone else doing well, you see that you might not make the team straight away, then there are many small things advice. Then you have to go in order and process each and every thought and get done with them, instead of it becoming a black hole with a lot of negativities.

The clearest finding was the players use of their experience and career, since it has made them more conscious of their own mental processes and how it has impacted their performance. Similarly, research has indicated that older players can better handle a mentally tough load, and that a conscious relationship to these processes also indicate a bigger likelihood of handling weak performance (Saward et al., 2020). Tommy states:

Tommy: It is something that has come in recent years for me. I have simply become more observant of it. In recent years, I have been trying to think about it. Because when I was younger it was just like ‘yeah, then I got it, screw it and do not think about it’, but then it got to me a bit more than I thought. So now, if I’ve been taken out of the team for a game, I have to process the thought of why, and this and that, and finish that thought and then move on, instead of carrying it on to match day and all of next week.

### 3.2 Mental toughness

All players unanimously mentioned the importance of mental toughness and considered it to have the most direct impact on their performance, especially during games:

Chris: The most important thing for me is when I’m playing and in the heat of the game, to be strong mentally. You see, for example, Haaland, he is perhaps the world’s toughness player when it comes to adjusting on the pitch, and constantly chasing the next. If he misses a chance, he knows that another one will come very soon.

The players describe mental toughness as partly being rational in how they should face the challenge and focus on the things they can impact, and looking ahead in time on the challenges to come.

Frank: In a way, I have a kind of attitude that if you dare to stand in it and believe in what you are doing, then you will get what you deserve in the end. Then usually comes the reward too, so for me it has been an important thing to just stick with it and not be too impatient.

The players mainly talk about mental toughness as the ability to handle adversity by being patient and believing in themselves. As prior research indicates, they still relate mental toughness to self-confidence (Machida et al., 2017; Vealey and Chase, 2008). Tommy describes using inner dialogue as a tool to gain self-confidence to allow them to be mentally tough and highlights the relationship between mental processes (Liew et al., 2019; Williams et al., 2020).

Tommy: I know what I can do and I’m better than my opponent if I meet someone in position on the court. I do not know if I’m fooling myself or if I’m actually better, but in my head, I am, I honestly believe I’m better than him.

To be able to perform, the players talked about their individual performance and how they were tested mentally in periods. A player, Oscar, stated, ‘It is perhaps particularly important to have these types of things during the periods when you are being tested a bit, if you are a bit in and out (of the team), or if you encounter a lot of adversity’. They also highlight the importance of social–emotional support via teammates and their mentality when the team faced challenges as a team.

Oliver: In a way, people in slightly bad times just distance themselves from the project and the team and want to have less to do with it compared to when things are going well, because then everyone wants to be part of it. So being in emotional balance and creating energy and trying to support those around you in bad times, that’s also an important phase then.

As highlighted was age, experience, and family situation understood to impact their mental toughness. One of the players, Chriss, described how a bad involvement in a game affected the next game at an earlier age, while they had now become better at readjusting according to the situation.

Chris: In the past if I made a mistake or lost a duel or lost a match or something, it could affect me for a long time then, and maybe become a little destructive. Now I think that I am much better at adjusting and thinking about the next situation, the next training session, the next match, and I think that will perhaps come a little with experience and age.

Players noted how they learn through experience by reflecting on their own performances (DeWiggins et al., 2010). For instance, when they were younger, they could see what was needed but still considered it hard to do this right thing. Others also highlighted that they gained a new perspective when they became a parent, as they had to change focus and let go of some thoughts when they got back to their family after the game or training. For instance, Chris stated, ‘I now have a child that I come home to, which means that when I come in the door now, I have to readjust and think about other things’.

### 3.3 Arousal regulation

Players highlighted a need for arousal regulation before the match. They arousal regulation as a part of their mental process and it impacts them in a positive way if used wisely, since too much energy might have a negative impact:

Frank: Before then I was very much like looking forward to the match and you kind of walk around waiting for the match to start, and that was actually one of the first things I brought up with the mental coach.

When talking about their thoughts during games, players highlighted the need to be focused and not let the mind wonder away, and stay in the present. Oscar noted, ‘If you walk around thinking about too many other things, then you have not found the right tension level’. Meanwhile, others talked about situations where they needed to adjust their arousal either up or down.

Oliver: Coming to the stadium in the city, it was like ‘here I have to work on myself’, compared to playing an eight o’clock match in the stadium in front of a number of spectators with floodlights, I actually feel that. And the feeling you get is that ‘here you get free energy and excitement’.

As a part of their arousal regulation, some players, such as Chris, used self-talk as an unconscious and automated mechanism to not open the mind to impressions in pointed and important moments (Beilock and Gray, 2007). Self-talk is used, for example, to avoid a high degree of tension from actors in the external environment. Here, an automated process is clearly initiated in which one thinks step by step; that is, it is an unconscious awareness of how one solves difficult situations by making the necessary adjustments required by the situation (Beilock and Gray, 2007).

Still, we also found individual variations during matches, where some players are conscious of their use of self-talk as a tension-regulating technique:

Oscar: The way I work, I work a lot with the way I talk to myself and about myself. And there is a lot to do with self-confidence, if you are able to describe yourself well, and tell yourself that you are good, then you will also perform better.

### 3.4 Self-confidence

Self-confidence was considered to impact performance (Knight et al., 2017), especially in decisive situations during a match (Machida et al., 2017):

Peter: After all, you get tremendous self-confidence from good performances. It’s actually what gives me the most confidence. The more good things you do, the better you play.

Furthermore, players perceive a relationship between self-confidence and performance, as highlighted by research (Hwang et al., 2017). Often, older and more experienced players are expected to be self-confident. Still, players consider self-confidence to also be important early in the career:

Oliver: Confidence is something that is… it’s not something that’s permanent, it’s fleeting. And that is independent of whether you are 15 or 30 years old. I’ve felt a lot of that kind of security in the last 10 seasons, and then I kind of get it NOW, when I’m old (30–35) and feel like I’m like ‘damn, I’m 15 years old and insecure and that bit’. In a way it’s kind of nice to feel that. It is perhaps as much about the fact that self-confidence is something that is not permanent then, that you have become more experienced and older, because self-confidence is a fresh commodity.

Thus, self-esteem is highlighted as a phenomenon that can occur regardless of age and experience (Knight et al., 2017). Peter points to this, stating “In the past I could think, ‘Shit, I cannot do that, because I’m not very good at it,’ and then you usually fail when you have that mindset”. As the players also highlight, self-confidence can come from other parts of their lives (i.e., education). Oliver believes that it is also important to master other arenas to avoid that everyday life as a footballer becoming all-consuming and highlighting the value of studying, ‘In the current period, education gives me a lot, that you have another arena to feel mastery in’. He also highlighted how the lack of mastery during injury periods negatively affected his self-confidence, ‘The reason why I have little self-confidence after periods of injury, for example, I know it’s about the fact that I have not had recent experiences where I’ve somehow mastered situations that have given me self-confidence’. Others experienced the opposite when they got injured as no longer have the pressure to perform, which positively affects their self-confidence:

Peter: What’s a bit funny actually, is that when I’m injured, that’s perhaps when I gain the most confidence. Then you get a bit out of the football bubble, you kind of do not have to perform every day, and then you kind of get to reset a bit.

In this professional football context, the fear of failure could overcome the joy of success (Coulter et al., 2010). Then, self-talk and inner dialogue can be used as a preventive tool by using affirmations that create security and self-confidence, as Peter tells (Konter et al., 2019). Such affirmations are exemplified, as S1 notes, as a tool to overcome negative thinking; or, as Frank highlights, relate self-confidence to self-image, which can be the basis for a solid self-confidence.

Tommy: If you first start to doubt, then it’s about being able to reset a bit, and put your finger in the ground and think ‘where am I?’. Yes, I play at a club and I’m here for a reason. There’s a reason why I’m here, I’m a good football player somehow.

Frank reflects on how both well-being and one’s qualities impact self-confidence, and thus, performance. They describe the inherent feeling of feeling good off the pitch as an important premise for performing on the pitch. Peter also refers to this inherent self-confidence as a strong desire, ‘[I do not] want good performances on the field to give me good self-confidence. I’d rather have an automatic self-confidence elsewhere in life that also makes me confident on the pitch’. However, although self-esteem is shown to be a dynamic phenomenon, self-image emerges as the most fundamental source of continuous self-esteem.

Another way of impacting the players’ self-confidence is the use of videos and visualisation, which many players describe as useful to boost their self-confidence. Oscar notes, ‘Maybe watch a video of things I’ve done, so for me it works then, in periods where I feel like I’m not mastering everything (Tommy) and: I like to watch videos of myself succeeding’. In a professional football context, mental skills can divide the professional players from the non-professional ones (Coulter et al., 2010). This also highlights the multidimensional aspect, since mental processes not only impact performance, but that also other mental processes.

### 3.5 Players’ use of a sport psychologist and their impact on performance

Players described a wish to have access to a sport psychologist in their own club, consistent with research on players in English professional football (Nesti, 2010). However, players’ actual access to a sport psychologist differed (see Table 1):

Chris: We do not have anyone who is a permanent employee, but I could definitely imagine it. I think maybe in football you think that in a physical team or medical team, you have to have a club doctor and a physio, and maybe a physical coach, but I do not think you often think about how important it could be to have a mental coach as well.

As Chris stated, the sport psychologist introduced new specific techniques unknown to them before. Peter stated, ‘It is the mental coach who have given me such techniques’, which they describe further as follows:

Peter: Out on the pitch, I have learned from the mental coach that I should occasionally take a few breaks where I reset and breathe in, breathe out, in and out, in and out a few times to calm myself down on the pitch, and reset and be ready again.

Trust was an important issue for some players. Research shows that mental techniques, such as visualisation and meditation, are effective (Konter et al., 2019), which was highlighted by one player. Others specified how they have used meditation, mindfulness, arousal regulation, and inner dialogue to overcome difficulties.

Oscar: I have become better at being aware of the way you talk to yourself, how it can affect performance, and just letting go and you do not always have to perform at your best.

Still, some players had not been able to use these learned techniques, although they know they have been found to be effective:

Oliver: If there had been some specific techniques that had worked for performance development, then it would have been much easier. I feel that it gets a little too diffuse and a little too high hanging sometimes, things like visualisation and meditation and that bit. I know in a way that research says that it can be beneficial, but I have not been able to translate this knowledge into ‘oh, this I know works’ and this gives me better conditions to perform, I have not found that yet.

Others did not use the sport psychologist the team had access to. Instead, Tommy used their experience, stating, ‘I have good control over it on my own and have found a method that works well for me, and then I continue with it until I feel that it does not work anymore’. The player focused on former experiences and that this affected his performance in professional today.

Although the players had difference experiences with a sports psychologist, all players had experience with one or more during their career. For some players, the sports psychologist’s impact was related to periods with many matches and the frequent need to perform, or during injury periods. Meanwhile, others highlighted their general value and how other clubs had success with using one.

Chris: You see, for example, that they have done it in a rival club, and there they have paid tribute to the work of a sports psychologist. It is about percentages, and of course at the top level you are more dependent on margins. If you can get margins from having a mental coach then it’s worth it, but of course it also depends a lot on club finances and priorities and things like that.

Thus, the impact of the sports psychologists appears to be different from an individual or group perspective. Most players highlight the individual talks with the sport psychologist, while there was one exception, Tommy, who liked the team talks better than the individual talks.

Tommy: I think the presentations he had in front of the team were very nice. I liked them a lot, because then he generally talked about tricks and such, to get into battle mode as a team. It seemed to the group that they also thought it was good, but as a duo I struggled a bit, I did.

The players with experience from international football highlighted both the individual and team talks. They noted how the mental coach had helped them and the team to redirect the focus and handle especially the media attention. Furthermore, the mental coach also equipped them to cope with the increased tension when competing on a higher performance level.

Frank: We have been out in Europe a bit, where things are quite different from Norway, and where there is even more media and even more pressure on you from the outside as well. He the mental coach has been great there, he has come in and in a way talked about how we as a group and individuals can shut out disturbances.

Some players did reflect on why they did not have access to sports psychologist. Peter noted, ‘It’s the economy and all that, we cannot afford it. The money is rather spent on an extra player. I believe deep down that coach thinks he is a good sports psychologist even then’. Similarly, Chris, who belongs to the same club as Peter, said that they have received the same answers regarding the employment of a sports psychologist. Nesti (2010) highlighted the difficulties of convincing coaches and the club management to prioritise this when the economic aspect is often the issue. Oliver highlighted that this was out of their control:

Oliver: I think it is the knowledge that is lacking a lot, and then I think that those in the club management today also have their own preferences. They are part of a football that was played years ago. So, the mental part was not even relevant (for them). When I talk about macho culture in our time, the culture they were a part of was really a proper macho culture.

Some highlighted the importance of the introduction of a sport psychologist at an early age. Peter stated, ‘I think if I had not gone to a mental coach when I was 20 and learned a little bit more about it, I think I’d have hit the wall, I’m pretty sure’. These results show that the players used both concrete mental techniques learned by a sport psychologist and more general techniques during their careers which they have learned to use themselves. This is in line with earlier research stating that sport psychological interventions are effective on players’ ability to handle stress (Miçoogullari and Ekmekçi, 2017; Nesti, 2010).

## 4 Discussion

We explored professional footballers’ perceptions and experiences around the relationship between mental processes and performance, especially those related to mental characteristics such as arousal regulation, mental toughness, and self-confidence. Furthermore, we explored players’ perception and experience of a common language related to mental skills and processes while working with a sports psychologist both in their current and earlier clubs.

The findings show that the youngest players seem to be most aware of the use of a sports psychologist. This may be because these players reach senior level earlier, which requires more of them mentally (i.e., mental toughness) than older players (Saward et al., 2020; Williams et al., 2020). Mental processes have been shown to be important for performing well (Saward et al., 2020), especially with respect to arousal regulation, mental toughness, and self-confidence. Players highlighted mental toughness as the most important mental process for their performance. This is because it is perceived as a fundamental mental quality for handling challenging situations before, during, and after matches (Thelwell et al., 2005; Wieser and Thiel, 2014). However, mental toughness was also considered a product of age and experience. Older players tend to have experienced more situations that enable them to handle adversity better than younger players. We also illustrated how self-confidence directly impacts on-field performance. This is consistent with the literature, which shows that compared with players with low self-esteem, a soccer player with high self-confidence will have greater faith in their own skills, and thus, will be more willing to take chances and challenges on the pitch (Coulter et al., 2010). Although the professional football context is an arena characterised by high performance, some players and clubs experience higher expectations than others. In this study, some players play in clubs which participate in major European tournaments, such as the Europa league, with higher external pressure and attention from the media. In such situation, using active mental training can increase mental toughness, and thus, players’ performances (Miçoogullari and Ekmekçi, 2017).

The players’ reflections revealed a desire and a need to work in a more structured way on the development of their mental skills in consultation with sports psychologists both as individual players and as a group of players (Nesti, 2010). Since the players related age and experience as two of the most decisive factors for the players’ mental toughness, the lack of references (age or experience) reinforces the need for a sports psychologist to be able to handle the situation such that they contribute to increasing the player’s and team’s performance. Still, a foundation of trust is needed between the player and sports psychologist (Konter et al., 2019), especially in the professional football context because of the performance culture and need for short term results. Consequently, sports psychologists need to deliver visible results. This is part of the reason why several coaches and clubs are sceptical about employing sports psychologists because it is difficult to collect objective data (McDougall et al., 2015; Feddersen et al., 2023). No experience or perception will be interpreted the same by two players because of individual differences arising not only from genetics and the environment but also from their thinking (Nesti, 2010).

Langagergaard (2017) argued that the context of performance differs compared to other contexts, and may be similar to the context of the military or police. Furthermore, the author highlighted the need for establishing a common language to develop a good performance culture that the team can use to move in the same direction. In collaboration with a sports psychologist, if the club can create an interaction between this individual complexity and the incorporation of a common language in the player group, one may argue that the effect will be greater and the performances will improve (Langagergaard, 2017). Although an individual player has developed good techniques for themself or in consultation with a sports psychologist, one’s own mental processes can prevent the full potential of the group of players, for example, by allowing their confidence to influence the outcome of situations in a match in a way that is disadvantageous to the player. Nevertheless, one can argue that there are reasons why one-to-one conversations are so important because it takes time to establish relationships that influence mental training (Nesti, 2010).

### 4.1 Strengths and limitations

This study does not distinguish between the players perceptions of mental coaches and sport psychologists, which is a limitation. Furthermore, mental coaches, who often have experience from the sports context, substantially differ from sports psychologists, who have an academic background from the field of psychology. Another limitation was that two of the authors knew the participants which also was part of the reason as why the players was recruited to the study. This might mean that they were biased to have a positive attitude towards both participating, but also to the topic of interest in the study. Such a strategic sample might mean that other players might have other experiences compared to this sample.

### 4.2 Future research

Future studies should include the actors related to players in the performance context of professional football, since their impact on players’ performance is essential. Furthermore, case studies or similar approaches with follow-up or longitudinal studies can be conducted with the same players as they get older, and more experienced both as football players and with the field of sport psychology.

In the professional football context, players have many actors and stakeholder around them. Players often find it difficult to know which of these stakeholders are introducing stability and conformity in their everyday life. These actors/stakeholders are trying to impact them, and both directly and indirectly impact their performance. Future studies should through both qualitative and quantitative studies focus on how the players differ in their needs and how the surrounding set-up impacts their performance, as their performance is matches and training are related. Another area worth exploring is the degree to which their well-being is related to their ability to perform. Most studies have focused on junior football, whose context differs from that of performance and elite football. As such, more studies are needed in the latter context.

## 5 Conclusion

This study shows that mental processes are important for performing (Saward et al., 2020), especially those related to arousal regulation, mental toughness, and self-confidence. Players highlighted mental toughness as the most important mental process for their performance because it is perceived as a fundamental mental quality for handling challenging situations before, during, and after matches (Thelwell et al., 2005; Wieser and Thiel, 2014). However, mental toughness was also considered a product of age and experience. Older players tend to have experience of more situations that enable them to handle adversity better than younger players. Interestingly, the youngest players seem to be most aware of the use of a sports psychologist. This may be because these players reached the senior level earlier, which requires more of them mentally (i.e., mental toughness) than older players (Saward et al., 2020; Williams et al., 2020). This study indicates that coaches should focus on developing the players mental toughness and especially among the younger players in professional football since this study show that older players handle adversity better based on experience. Still, since the younger players had a more aware use of a sport psychologist, it might be that today’s younger players has learned these skills to be able to keep up a career in professional football.

## Data Availability

The raw data supporting the conclusions of this article will be made available by the authors, without undue reservation.

## Appendix

APPENDIX 1: Interview guide.

**Opening questions**

- Can you tell us a little about yourself?
  - Interests
  - Education
  - Job
- Can you briefly describe your football career?
  - Clubs
  - Circuit layers
  - National team
  - Role in respective teams

**Theme 1: Skill development in football**

- What skills do you think are required to take the step to become a professional footballer?
  - Technical factors
  - Social factors
  - Psychological factors
  - Physical factors
- Do you feel that some skills are more important than others for your development as a player?

**Theme 2: Mental processes**

- What do the words mental processes mean to you?
  - In everyday life
  - In football

**(Interviewer defines mental processes).**

- Do you consciously and actively use mental processes in your everyday training and competition?
  - Which – any special techniques?
- Where did you possibly learn or hear about these?
  - Alone or as a club
  - During battle
  - Mental toughness – own definition followed by common definition
  - Self-regulation – own definition followed by common definition
  - Voltage regulating techniques
  - Self-confidence – own definition followed by shared definition
- Others?
- Do you think that mental processes are important for the level you play at today?
  - Which ones?
  - In what way?
- Which mental skills do you think are most important to have as a footballer?
- When is it most important for you to be aware of mental processes?
- Injuries
  - Out of the team
  - Fixed on the team
  - Training
  - Struggle
  - Media
- What/who is most important to your development/use of mental processes?
  - Yourself
  - The coach
  - Family and friends
  - The player group
  - Support device
  - Other?
- Are mental processes something you spend time talking about in the player group?
  - Consciously/unconsciously

**Theme 3: The relationship between mental processes and performance**

- How do you experience the relationship between mental processes and performance in your training everyday competition?
  - Are you aware of whether the mental affects your performance?
- If you/you have had a mental coach - how can that person help with improve your mental skills?
  - Have you had a response or benefit from this training?
- Does your role in the team have an impact on how the mind affects your performance?
  - Age
  - Experience
  - In and out of the team
  - Injuries
- If you have played in several clubs and teams; have you experienced differences in how mental processes affect your performance based on where you are and who you are with?
  - Age-specific teams
  - Circuit team/national team
  - A-team/B-team
  - Various leagues
  - Different player groups
  - Different trainers
  - Have you worked differently in the clubs with mental processes?

**Closing question**

- Something you would like to add about the topic that you have not been told in this interview?

## References

[ref1] Beilock S. L. Gray R. (2007). “Why do athletes choke under pressure?” in Handbook of sport psychology (3. Utg.). eds. Tenenbaum G. Eklund R. C. (Wiley), 425–444.

[ref2] Benítez-Sillero J. D. D. Martínez-Aranda L. M. Sanz-Matesanz M. Domínguez Escribano M. (2021). Determining factors of psychological performance and differences among age categories in youth football players. Sustain. For. 13:7713. doi: 10.3390/su13147713

[ref3] Braun V. Clarke V. (2006). Using thematic analysis in psychology. Qual. Res. Psychol. 3, 77–101. doi: 10.1191/1478088706qp063oa

[ref4] Braun V. Clarke V. Terry G. Hayfield N. (2019). “Thematic analysis” in I handbook of research methods in health and social sciences. ed. Liamputtong P. (Springer), 843–860.

[ref5] Brinkmann S. Kvale S. (2018). Doing interviews. 2nd Edn: SAGE Publications Inc.

[ref6] Coulter T. J. Mallett C. J. Gucciardi D. F. (2010). Understanding mental toughness in Australian soccer: perceptions of players, parents, and coaches. J. Sports Sci. 28, 699–716. doi: 10.1080/02640411003734085, PMID: 20496223

[ref7] Coulter T. J. Thelwell R. C. (2019). “Mental toughness development in football” in Football psychology. (Eds.) E. Konter, J. Beckmann, and T. M. Loughead. (Routledge), 23–37.

[ref8] DeWiggins S. Hite B. Alston V. (2010). Personal performance plan: application of mental skills training to real-world military tasks. J. Appl. Sport Psychol. 22, 458–473. doi: 10.1080/10413200.2010.500606

[ref9] Dodd K. D. Newans T. J. (2018). Talent identification for soccer: physiological aspects. J. Sci. Med. Sport 21, 1073–1078. doi: 10.1016/j.jsams.2018.01.009 29789264

[ref10] Feddersen N. B. Champ F. Sæther S. A. Littlewood M. (2023). How psychologists in Men’s English football academies evaluate their working context and adopt an appropriate professional practice framework. Sport Psychol. 37, 170–178. doi: 10.1123/tsp.2022-0154

[ref11] Forsman H. Blomqvist M. Davids K. Liukkonen J. Konttinen N. (2016). Identifying technical, physiological, tactical and psychological characteristics that contribute to career progression in soccer. Int. J. Sports Sci. Coach. 11, 505–513. doi: 10.1177/1747954116655051

[ref12] Gould D. Dieffenbach K. Moffett A. (2002). Psychological characteristics and their development in Olympic champions. J. Appl. Sport Psychol. 14:172204, 172–204. doi: 10.1080/10413200290103482

[ref13] Hwang S. Machida M. Choi Y. (2017). The effect of peer interaction on sport confidence and achievement goal orientation in youth sport. Soc. Behav. Personal. Int. J. 45, 1007–1018. doi: 10.2224/sbp.6149

[ref14] Ivarsson A. Kilhage-Persson A. Martindale R. Priestley D. Huijgen B. Ardern C.. (2020). Psychological factors and future performance of football players: a systematic review with meta-analysis. J. Sci. Med. Sport 23, 415–420. doi: 10.1016/j.jsams.2019.10.021, PMID: 31753742

[ref15] Jones G. Hanton S. Connaughton D. (2002). What is this thing called mental toughness? An investigation of elite sport performers. J. Appl. Sport Psychol. 14, 205–218. doi: 10.1080/10413200290103509

[ref16] Jordet G. (2019). “Working with psychology in professional football” in Routledge handbook of elite sport performance. (Eds.) Collins A. Cruickshank and G. Jordet (red.), (Routledge), 114–132.

[ref17] Knight C. J. Harwood C. G. Gould D. (2017). An introduction to sport psychology for young athletes. In Sport psychology for young athletes, (Routledge), 1–6.

[ref18] Konter E. Beckmann J. Mallett C. J. (2019). “Psychological skills for football players” in Football psychology. (Eds.) Erkut K., Jürgen B., and Todd M. L. (Abingdon, Oxon, United Kingdom: Routledge), 179–197.

[ref19] Kremer P. J. Marchant D. B. (2002). Reflections and considerations of providing sport psychology services with professional football players. Sci. Football IV, 294–299.

[ref20] Langagergaard M. (2017). Performance psychology: handbook for Nordic counter terrorism units: Scandinavian Book A/S.

[ref21] Liew G. C. Kuan G. Chin N. S. Hashim H. A. (2019). Mental toughness in sport. Ger. J. Exerc. Sport Res. 49, 381–394. doi: 10.1007/s12662-019-00603-3

[ref22] Machida M. Otten M. Michelle T. M. Vealey R. S. Ward R. M. (2017). Examining multidimensional sport-confidence in athletes and non-athlete sport performers. J. Sports Sci. 35, 410–418. doi: 10.1080/02640414.2016.1167934 27086937

[ref23] MacNamara Á. Button A. Collins D. (2010). The role of psychological characteristics in facilitating the pathway to elite performance part 1: identifying mental skills and behaviors. Sport Psychol. 24, 52–73. doi: 10.1123/tsp.24.1.52

[ref24] MacNamara Á. Collins D. (2011). Development and initial validation of the psychological characteristics of developing excellence questionnaire. J. Sports Sci. 29, 1273–1286. doi: 10.1080/02640414.2011.589468, PMID: 21812724

[ref25] Markula P. Silk M. L. (2011). Paradigmatic approaches to physical culture. London: Palgrave Macmillian.

[ref26] McDougall M. Nesti M. Richardson D. (2015). The challenges of sport psychology delivery in elite and professional sport: reflections from experienced sport psychologists. Sport Psychol. 29, 265–277. doi: 10.1123/tsp.2014-0081

[ref27] Miçoogullari B. O. Ekmekçi R. (2017). Evaluation of a psychological skill training program on mental toughness and psychological wellbeing for professional soccer players. Univ. J. Educ. Res. 5, 2312–2319. doi: 10.13189/ujer.2017.051222

[ref28] Mitchell T. O. Nesti M. Richardson D. Midgley A. W. Eubank M. Littlewood M. (2014). Exploring athletic identity in elite-level English youth football: a cross-sectional approach. J. Sports Sci. 32, 1294–1299. doi: 10.1080/02640414.2014.898855 24786769

[ref29] Mitic P. Nedeljkovic J. Bojanic Ž. Franceško M. Milovanovic I. Bianco A.. (2021). Differences in the psychological profiles of elite and non-elite athletes. Front. Psychol. 12:635651. doi: 10.3389/fpsyg.2021.635651, PMID: 33815222 PMC8012685

[ref30] Nesti M. (2010). Psychology in football: Working with elite and professional players. Routledge.

[ref31] Rye A. Ransom D. Littlewood M. Sæther S. A. (2022). Performance and organizational stressors in the junior-to-senior transition in football. Curr. Issues Sport Sci. 7:3. doi: 10.36950/2022ciss003

[ref32] Saward C. Morris J. G. Nevill M. E. Minniti A. M. Sunderland C. (2020). Psychological characteristics of developing excellence in elite youth football players in English professional academies. J. Sports Sci. 38, 1380–1386. doi: 10.1080/02640414.2019.1676526, PMID: 31607218

[ref33] Thelwell R. Weston N. Greenlees I. (2005). Defining and understanding mental toughness within soccer. J. Appl. Sport Psychol. 17, 326–332. doi: 10.1080/10413200500313636

[ref34] Vealey R. S. Chase M. A. (2008). “Self-confidence in sport: conceptual and research advances” in Advances in sport psychology. ed. Horn T. S. (Champaign, IL: Human Kinetics), 65–97.

[ref35] Wahyuni D. (2012). The research design maze: understanding paradigms, cases, methods and methodologies. J. Appl. Manag. Account. Res. 10, 69–80.

[ref36] Wieser R. Thiel H. (2014). A survey of “mental hardiness” and “mental toughness” in professional male football players. Chirop. Manual Therap. 22, 1–6. doi: 10.1186/2045-709X-22-17 PMC4062893 24735867

[ref37] Williams A. M. Ford P. R. Drust B. (2020). Talent identification and development in soccer since the millennium. J. Sports Sci. 38, 1199–1210. doi: 10.1080/02640414.2020.1766647 32568000

